# Single-Center Cohort of Pediatric Patients with High-Risk Neuroblastoma Receiving Immunotherapy

**DOI:** 10.3390/cancers17111824

**Published:** 2025-05-30

**Authors:** Emese Zsigrai, Sándor Barna, Zsuzsanna Gaál, Lilla Macsi, István Szegedi, Miklós Petrás, Csongor Kiss

**Affiliations:** 1 Division of Pediatric Hematology-Oncology, Department of Pediatrics, Faculty of Medicine, University of Debrecen, 4032 Debrecen, Hungarymikey@med.unideb.hu (M.P.); 2 Nuclear Medicine, Medical Imaging Clinic, Faculty of Medicine, University of Debrecen, 4032 Debrecen, Hungary

**Keywords:** children, neuroblastoma, dinutuximab beta, nivolumab

## Abstract

Background: Neuroblastoma (NB) is one of the most common solid tumors in children, still showing a high mortality rate despite recent advances in therapy. A recent breakthrough was the introduction of Dinutuximab beta, yielding further improvements in survival. Dinutuximab beta is an anti-GD2 monoclonal antibody that targets GD2 expressed on the cell surface of neuroblastoma cells. Evidence suggests that Dinutuximab beta combined with Nivolumab may offer an effective synergistic treatment approach. Methods: In our center, immunotherapy was introduced in 2021 as part of maintenance treatment. The aim of this retrospective study was to analyze our data with a focus on the response, side effect profile and tolerability of Dinutuximab beta in HR and relapsed or refractory (r/r) NB. Results: Between 2021 and 2024, we treated 15 patients with neuroblastoma. Twelve patients had high-risk disease, of whom five received Dinutuximab beta as part of maintenance treatment according to protocol HR-NBL 1.8/SIOPEN. Two patients achieved complete remission after immunotherapy. One achieved long-lasting remission, while another relapsed. Three patients with inoperable tumors developed a partial response, but they relapsed and were diagnosed with metastases later. These patients subsequently initiated treatment with Temozolomide + Irinotecan in combination with Dinutuximab beta and also with Nivolumab as a relapse protocol. Therapeutic responses were assessed by the imaging, pathology and flow cytometry analysis of bone marrow. Apart from one complication (hypotension as part of capillary leak syndrome) subsiding spontaneously, no other severe adverse events were observed. Conclusions: Our experiences confirm that immunotherapy, including Dinutuximab beta and Nivolumab, is safe and well tolerated. The standardization of the application of Dinutuximab beta and in combination with novel therapeutic agents in maintenance and refractory/relapsed cases may contribute to improved treatment outcome results.

## Simple Summary

High-risk neuroblastoma (NB) still has a high mortality rate despite advancements in therapy. The introduction of Dinutuximab beta has yielded further improvements in survival. The latest data prove that combining immune checkpoint inhibitors show an additional contribution to a more successful therapeutic response. Here, we report a retrospective study on a small institutional cohort of high-risk neuroblastoma patients treated with Dinutuximab beta and Nivolumab in relapsed or refractory (r/r) cases (2021–2024). Of twelve patients, five were administered Dinutuximab beta and three were given Nivolumab in combination due to a residual mass still being present at the end of treatment. Two patients achieved remission, one remains in this state, and another relapsed in 4 months after concluding treatment. The other three patients—with an inoperable residual mass—had partial response while undergoing Nivolumab therapy. Apart from one serious complication resolving spontaneously, the use of Dinutuximab beta was well tolerated. Our results underline the safe use of Dinutuximab beta and also prove its good therapeutic response in an r/r setting.

## 1. Introduction

Neuroblastoma is a malignant tumor originating from immature embryonic neural crest cells, known as neuroblasts, that give rise to the sympathetic nervous system. It most commonly develops in the adrenal medulla; in other cases, it can appear paravertebrally along the spine. It is the most frequent extracranial solid tumor of childhood, affecting mainly children under 5 years of age, with 25% of cases occurring before the age of 1 year. In Hungary, there are about 15–20 new pediatric cases diagnosed annually [1,2].

The development of treatment for pediatric neuroblastoma has evolved through several key stages, with advancements made in understanding the disease, improving survival rates, and minimizing side effects. Until the introduction of chemotherapy, especially combined chemotherapy (i.e., cyclophosphamide, vincristine, platinum derivatives) from the 1970s, the overall survival was dismal and the majority of the patients continued to fare very poorly.

Based on several features (incl. age, stage, histology, *MYCN* status, and tumor cell ploidy), neuroblastoma can be classified into three different risk groups—i.e., low, intermediate and high (LR, IR and HR groups). In parallel with improvements in imaging and laboratory techniques—such as the ^123/131^I-meta-iodobenzylguanidine scan (mIBG), histology according to the Shimada classification, the DNA index, *MYCN* amplification and other genotypic patterns—the implementation of more personalized, risk-tailored strategies were set out and established. According to the risk classification, treatment currently encompasses a wide spectrum, ranging from simple observation to surgical intervention and radiotherapy, as well as high-dose chemotherapy, autologous bone marrow transplantation, and the use of immunotherapy. LR patients may be treated with surgical resection only and show a 5-year EFS of 90.7% ± 1.1% and OS of 97.9% ± 0.5%, while IR patients treated with a short course of standard chemo- and supportive therapy also have an excellent prognosis, exhibiting a 5-year EFS of 85.1% ± 1.4% and OS of 95.8% ± 0.8%, as reported by the recently revised neuroblastoma risk classification system [3,4]. High-risk patients, however, still continue to have a disheartening prognosis with an overall survival rate of around ~50% despite recent advances in the treatment, accounting for at least 15% of death cases from malignant diseases in childhood [5]. The treatment course of HR patients consists of induction, consolidation, local therapy and maintenance therapy. Induction and consolidation are aimed at inducing remission and eradicating remaining tumor cells that may have survived induction, respectively [6]. The adjuvant radiation of the primary site and in certain cases of metastatic sites may also be indicated [7,8].

A recent significant breakthrough in the treatment of neuroblastoma was the introduction of Dinutuximab beta therapy. Dinutuximab beta is a chimeric monoclonal antibody designed to target the disialo-ganglioside 2 (GD2) molecules expressed on a neuroblastoma cell and induces cell lysis through antibody-dependent cell-mediated cytotoxicity and complement-dependent cytotoxicity. It was approved first by the FDA (US) in 2015 and its first approved indication was its application as part of maintenance therapy following induction, consolidation and local treatment in order to control minimal residual disease (MRD) [9]. It has also been approved by the European Medicines Agency for the treatment of high-risk (HR) NB in patients aged ≥ 12 months who have previously received induction chemotherapy and achieved at least a partial response, followed by myeloablative therapy and stem cell transplantation, as well as patients with a history of relapsed or refractory (r/r) NB, with or without residual disease. Most recently, however, the use of Dinutuximab beta has been extended for controlling bulky disease, either after the induction therapy of de novo cases not exhibiting a complete remission or in r/r disease. In the former cases, the use of Dinutuximab beta has been suggested in combination with the chemotherapeutic agents Temozolomide and Irinotecan, known as TEMIRI blocks. Thus, it has now also been included in the treatment protocol for HR patients, prior to autologous hematopoietic stem cell transplantation (HSCT) and when the extent of the minimal residual disease (MRD) is still unacceptable to perform an efficient HSCT [10]. Furthermore, recently, GD2 was targeted not only by a monoclonal antibody but also by GD2.CART cells, which is a promising experimental approach exhibiting its efficacy in clinical trials for the future treatment of patients with neuroblastoma [11]. In addition, increasing evidence supports the successful use of immune checkpoint inhibitors (ICIs; Nivolumab and Pembrolizumab) in r/r neuroblastoma. Recently, a combination of Dinutuximab beta with ICIs showed further improvements in patient survival data [12,13].

Since 2021, Dinutuximab beta has been used in the treatment of high-risk (HR) patients in Hungary, including in our institution, where we used Dinutuximab beta as part of maintenance therapy. We treated five patients with HR disease, and unfortunately, none of these patients entered complete remission after induction and consolidation. In each of the cases, Dinutuximab beta was used to control bulky residual disease, either at the primary tumor site or metastatic sites. In three cases, an insufficient response to Dinutuximab beta prompted us to include an ICI (Nivolumab) in the management of these patients. Nivolumab is a PD-1 inhibitor, which, according to the literature, exhibits a synergistic effect when combined with Dinutuximab beta therapy as was interestingly and recently shown in a murine model targeting the T-cell immunoreceptor with immunoglobulin and the ITIM domain (TIGIT) and PD-L1 with anti-GD2 immunotherapy [14]. Despite of the limited size of our institutional cohort, we think that these examples can serve as real-world evidence (RWE) supporting the beneficial potential of Dinutuximab beta alone or in combination with an ICI in HR, bulky neuroblastoma.

## 2. Material and Methods

We conducted a retrospective single-center case–cohort study on the use of Dinutuximab beta in the treatment of HR neuroblastoma patients in our center between 2021 and 2024. Patient data, clinical course during the treatment, relevant side effects, supportive therapeutic options and end-of-treatment outcomes are given and summarized. Characteristic imaging findings (MR, SPECT, and MIBG) are also shown. The patients and parents provided informed consent to the treatments. As Dinutuximab beta (Qarziba^®^, Recordati Netherlands B.V., Schiphol-Rijk, The Netherlands), has been approved for the use of treating HR neuroblastoma patients and bulky or r/r disease, ethical board approval was not necessary before starting the treatment. However, since the use of the immune checkpoint inhibitors—i.e., Nivolumab—has not yet been approved for the treatment of such patients, parental request and the permission of the National Center for Public Health and Pharmacy (NNGYK) was obtained and the drug (Opdivo®, Bristol-Myers Squibb Pharma EEIG, Dublin, Ireland) was provided by the National Health Insurance Fund of Hungary (NEAK).

Response to treatment was defined by protocol HR-NBL 1.8/SIOPEN. Complete response (CR) was defined as no tumor at primary or metastatic sites with normal catecholamine, very good partial response (VGPR) as a 90–99% decrease in the primary tumor with residual bone metastases assessed by a ^99^Tc scan, partial response (PR) as a >50% decrease in the primary tumor with a >50% decrease in metastatic sites including the number of bone and bone marrow sites, mixed response (MR) as a >50% decrease in any measurable lesions with a <50% decrease or <25% increase in any other existing lesions, no response (NR) as no new lesions, a <50% decrease and a <25% increase in any lesions and progressive disease (PD) as any new lesions or a >25% increase in existing lesions or new bone marrow involvement. Nivolumab treatment was initiated after receiving permissions from both the NNGYK and NEAK; 19 doses of 3 mg/kg/day infusion were administered every two weeks according to previous experiences with its promising and well-tolerated use in r/r pediatric solid tumors [15].

Dinutuximab beta treatment was performed according to protocol HR-NBL 1.8/SIOPEN; briefly, five cycles of Dinutuximab beta treatment were administered, lasting for 10 days, at a dose of 10 mg/m^2^/day or 0.33 mg/kg/day in the cases of children < 12 kg and >5 kg and 0.22 mg/kg/day in the cases of children < 5 kg. The total dose of Dinutuximab beta administered per cycle was 100 mg/m^2^, 3.3 mg/kg and 2.2 mg/kg, respectively.

## 3. Results

Between 2021 and 2024, 15 patients with histology-proven neuroblastoma were admitted to our institution. Twelve of these fifteen patients were diagnosed with high-risk (HR) disease. Two patients had *MYCN*-amplified tumors. The majority of patients were infants, with a median age of 1 year. There was a slight male predominance (Table 1).

**Figure 1:**
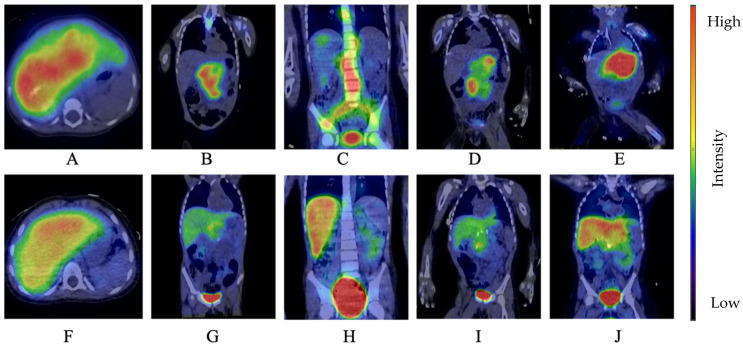
^123^I-mIBG scan of HR patients before (**A**–**E**) and after (**F**–**J**) 5 cycles of Dinutuximab beta therapy. (Patient 1 (**A**,**F**)—liver metastasis; Patient 2 (**B**,**G**)—retroperitoneal tumor, hepatic and retroperitoneal lymph node metastasis; Patient 3 (**C**,**H**)—paravertebral tumor; Patient 4 (**D**,**I**)—retroperitoneal neuroblastoma, paravertebral metastasis; Patient 5 (**E**,**J**)—retroperitoneal and mesenteric mass).

Of the 12 patients with HR NB, 5 patients received Dinutuximab beta treatment. The main clinical characteristics and outcome results of these patients are summarized in Table 2. Each of these patients had metastatic disease, including bone marrow involvement. Initial SPECT images of four patients are shown in Figure 1A–E. None of the patients reached complete remission before starting immunotherapy with Dinutuximab beta despite having undergone autologous HSCT (five patients) and local radiation therapy (four patients). Dinutuximab beta therapy was started to control bulky disease in these patients (measurable primary and/or metastatic-space occupying lesions (four cases) or bone marrow involvement (one case)). Each of these five patients received at least five cycles of Dinutuximab beta as suggested by protocol HR-NBL 1.8/SIOPEN. Having concluded five cycles, two patients entered complete remission as checked by a physical investigation and imaging techniques including ^123^I-mIBG SPECT/MR (Figure 1F–J). One patient developed partial response and two patients had stable disease.

Out of the two patients (Patients 1 and 3) exhibiting CR to Dinutuximab beta treatment, one has continuous complete remission (Patient 1) for over 24 months (Figure 1A,F). The second patient suffered a combined, multiple bone and bone marrow relapse (Figure 1C,H). In this case, a cycle of combined chemotherapy with Temozolomide and Irinotecan was given as salvage treatment, and permission for subsequent Dinutuximab beta therapy was sought from the NEAK; however, the parents refused further chemo-and immunotherapy and removed the patient from our institution to receive alternative treatment with dendritic cells. The patient returned with end-stage, progressive disease.

Three patients (Table 2, Patients 2, 4, and 5) with PR received further therapy. In these cases, immunotherapy was supplemented with an immune checkpoint inhibitor following five courses of Dinutuximab beta as per the protocol. Nivolumab was applied in combination with Etoposide. The first patient (Patient 2), who received Nivolumab treatment for a total of 19 cycles, did not show significant changes in tumor size. However, a new lesion with uncertain malignancy appeared in the lung, as discerned by chest CT. A PET-CT scan failed to clarify the origin of the space-occupying lesion, with both malignancy and inflammatory residue being considered as possible causes. The parents declined surgical sampling for histology. Follow-up imaging 4 months later showed an increase in the size of the lung lesion and the appearance of new liver metastasis and no change in the size of the primary tumor. Further treatment for refractory neuroblastoma is planned, including TEMIRI and Dinutuximab beta administration.

The second patient, who achieved PR after the first courses of Dinutuximab beta therapy according to the protocol (Patient 4), showed decreased activity in the retroperitoneal mass on an MIBG scan. Neither abdominal and cranial MRIs showed significant changes. Given the diagnosis of therapy-resistant neuroblastoma, treatment was augmented with Nivolumab. Despite this treatment, multiple new cerebral metastases developed; therefore, treatment was continued with a combination of TEMIRI and Dinutuximab beta. The patient is currently undergoing the fourth treatment cycle.

The last patient with PR (Patient 5) was the first to receive the initial five cycles of Dinutuximab beta combined with chemotherapy (Cyclophosphamide + Irinotecan). MIBG scan results confirmed a favorable therapeutic response. Treatment was planned to continue with chemotherapy in combination with the further application of Dinutuximab beta. However, the parents did not consent to this treatment plan, and adjuvant Nivolumab therapy alone was proposed instead. Subsequent imaging studies, however, indicated progression, and after further consultation with the parents, they eventually agreed to proceed with combined immunotherapy. Currently, the patient is receiving TEMIRI blocks, combined with Nivolumab and Dinutuximab beta. The latest imaging study described a residual tumor consistent with partial response.

Regarding safety and tolerability, the use of Dinutuximab beta was well tolerated. In accordance with the protocol, analgesics, antipyretics, and antihistamines were administered during the use of Dinutuximab beta. Pain management was initiated with continuous morphine infusion, along with a minor analgesic given every 4 h. Gabapentin was administered for the prevention of peripheral neuropathy and neuropathic pain. Side effects were graded according to the Common Terminology Criteria for Adverse Events (CTCAE v5.0, 2017).

The treatment of our first patient was conducted under intensive care unit conditions due to the anticipated side effects. This patient developed hypotension as part of capillary leak syndrome (Grade 3) during the first cycle and briefly required circulatory support (4 h), after which his symptoms rapidly improved, allowing anti-tumor treatment to continue. Alongside morphine analgesia, constipation (Grade 2) frequently occurred, and during the fourth cycle, subileus (Grade 2) developed. As a result, we switched pain management to Nalbuphine, which was used for subsequent cycles and in subsequent patients. In the case of further patients, we were able to gradually reduce continuous analgesia, and for the last patient, only occasional minor analgesics were used for pain control. In the other four patients, no therapy-related side effects or those requiring Dinutuximab beta suspension occurred in any cycle. Other mild or moderate side effects (Grade 1–2) that were observed included fever, edema, hypokalemia, and diarrhea. The treatments were generally well tolerated, and with the exception of the first case, all other treatments were conducted in our hematology ward without any special requirement.

## 4. Discussion

The treatment outcome results of HR neuroblastoma are still less than satisfying, presenting an unmet need for more effective therapeutic approaches. A major advancement in the neuroblastoma treatment was achieved in 2015 with the approval of Dinutuximab beta that has offered an important step forward to improving treatment results [16]. The findings of the pivotal phase III clinical trial of the Children’s Oncology Group (COG) was published in 2010; 226 children with HR neuroblastoma responding to initial treatment were investigated. The patients were randomized to receive either Isotretinoin alone or Isotretinoin and Dinutuximab beta in combination to control MRD. Two-year OS was 46% in the Isotretinoin-only arm and 66% in the patients receiving Dinutuximab beta [17]. The effect of immunotherapy with Dinutuximab beta was also proven by the high-risk neuroblastoma 1 trial (HR-NBL1 trial) of the International Society of Pediatric Oncology Europe Neuroblastoma Group SIOPEN; 466 patients did not receive immunotherapy, while 378 patients did, and the 5-year EFS/OS was 42%/50% and 57%/64%, respectively [18]. Ever since, a growing body of evidence has supported the superior efficacy of Dinutuximab beta for controlling MRD in neuroblastoma, so it has then been applied in the maintenance phase of the protocols in order to fight against minimal residual disease [19,20]. Recent studies of r/r neuroblastoma have shown the potential efficacy of Dinutuximab beta in this forlorn situation. Different approaches have been set out to increase the effectiveness of Dinutuximab beta (e.g., in combination with Temozolomide + Irinotecan or with Galunisertib by inhibiting TGF-beta1) that have led to improved outcomes compared to standard therapy in patients with HR neuroblastoma [21].

Due to the observed efficacy in the above scenario, the use of Dinutuximab beta has been extended to patients with bulky disease. In 2023, Wieczorek et al. retrospectively reviewed the clinical records of 54 patients with HR NB who received maintenance therapy with Dinutuximab beta in first-line treatment or r/r settings in Poland. They reported that 11 of 17 r/r patients (64.7%) had a complete response and the median OS was 33.1 months, with three-year PFS and OS at 0.75 and 0.86, respectively. Their findings demonstrated that treatment was generally well tolerated and that the use of Dinutuximab beta was feasible and beneficial as a first-line therapy and in r/r settings as well [22,23]. While the use of Dinutuximab beta in the treatment of neuroblastoma is well established and also licensed, the addition of Nivolumab is still experimental, with retrospective analyses and ongoing studies exploring the exact benefit of this approach [24,25,26,27]. It has been revealed that anti-GD2 antibody-mediated effects upregulate inhibitory immune checkpoint expression, and the combination of anti-GD2 treatment with an ICI results in synergistic treatment effects [28]. In an ongoing clinical trial of MINIVAN, Dinutuximab beta has been used in association with Nivolumab after 131-I mIBG therapy, with promising preliminary results [12]. This therapy is currently being administered in a compassionate care setting [29], and our case–cohort study may contribute as one of the first sources of real-world data for the upcoming evaluation of its outcomes.

It is noteworthy, however, that Mora and colleagues reported particularly good and favorable survival outcomes with the combined administration of Naxitamab, a GD2 antibody, and GM-CSF without PD-L1 blockade in a recently published phase II clinical trial involving 76 neuroblastoma patients. The study evaluated the efficacy of this combination therapy and observed an even superior efficacy, as evidenced by an overall survival rate of 93% and progression-free survival rate of 35% after one year [30]. In Hungary, unfortunately, neither of these two drugs are marketed and available as a treatment option. In our case–cohort study, based on the promising results with the combination of Dinutuximab beta and immune checkpoint inhibitors in r/r NB patients published by Ehlert and colleagues [13], Nivolumab was added to the treatment, aiming at helping to control the disease and sustaining SD status further on.

However, clinical data, especially real-world evidence, are still limited. This is why we report on a small cohort of HR neuroblastoma patients with bulky residual disease. Dinutuximab beta was applied in the course of the maintenance treatment and resulted in a complete response in the cases of two patients, while PR could have been achieved in three patients. One patient is still in long-lasting remission (for 24+ months to date). Since recent studies have suggested the feasibility and efficacy of targeting the PD1-PDL1 signaling pathway, we initiated the administration of Nivolumab as a continuation of treatment in our patients who showed partial remission following maintenance therapy [24,25,26,27]. Nivolumab was administered after treatment with etoposide, considering that recent results have shown that chemotherapeutic agents significantly increase the expression of stress ligands and inhibitory checkpoint ligands, i.e., GD2 and PDL1, as well [31]. One of the patients (N^o^ 2) was given Nivolumab in association with etoposide without Dinutuximab beta for 19 cycles before further Dinutuximab beta treatment was approved by the National Center for Public Health and Pharmacy and the National Health Insurance Fund of Hungary. At this point, the patient had gross residual disease (see Table 2), and Nivolumab alone did not result in a significant reduction in the residual tumor mass. Therefore, upon approval, Dinutuximab beta was added to the treatment of this patient, which she is currently receiving. Now, Nivolumab treatments are still ongoing in these patients; therefore, its efficacy cannot yet be judged. In the future, comparative analyses of studies employing similar therapeutic formulas will be warranted to elucidate the efficacy of this specific modality.

Of the five patients treated with Dinutuximab beta, two achieved remission, and one of them is still in this state, with long-lasting CR at 24 months after the end of treatment. Unfortunately, the other one relapsed 4 months after ending the treatment with Dinutuximab beta but refused further treatment and went to another institution to receive alternative medical care. Regarding the other three patients—with an inoperable residual abdominal mass—a partial response and stable disease (SD) status could have been achieved. Even more limited is the number of patients receiving Nivolumab monotherapy without Dinutuximab beta. In fact, we had only one such patient, who exhibited stable disease during the administration of Dinutuximab beta and Nivolumab combination, but we cannot draw definitive conclusions from this single case. Nonetheless, alongside the benefits of this therapeutic approach, the potential risks must also be emphasized, and close monitoring is required both during and after treatment. ICI (i.e., Nivolumab) treatment is associated with well-known side effects (skin toxicity, immune-related endocrinopathies like thyroid gland disorders or type 1 diabetes mellitus, gastrointestinal toxicity and hepatotoxicity, pneumonitis and, in rare cases, neurotoxicity, cardiac, ocular, renal and hematological (AIHA) disturbances) that must be continuously monitored to ensure their early detection and appropriate management, thereby supporting long-term survival with the highest possible quality of life for patients [32,33,34]. Further efforts are needed that focus on evaluating treatments with comparable therapeutic profiles to clarify the effectiveness of this particular approach.

Our results underlie that Dinutuximab beta is safe and well tolerated in patients with high-risk neuroblastoma, as established previously in multiple clinical trials, which is currently the standard of care for high-risk disease in the maintenance phase [9]. However, the application of Dinutuximab beta has not yet become everyday practice in patients with gross residual or recurrent neuroblastoma, particularly combined with Nivolumab. Therefore, we hope that our real-world data, even if only to a small extent, will actively contribute to the evaluation of therapies using this combination—an evaluation that is urgently needed to improve the still unfortunately poor survival rates of patients with HR, bulky, metastatic or r/r neuroblastoma. In light of these considerations, conducting continued research and further clinical trials with this combination therapy are of critical significance and are needed to improve survival rates and minimize the impact of treatment on long-term quality of life.

## 5. Conclusions

The treatment of pediatric neuroblastoma has progressed from basic surgery and radiation to a more comprehensive, multi-modal approach, integrating chemotherapy, targeted therapies, immunotherapy, and personalized medicine. A recent breakthrough in the treatment was the administration of Dinutuximab beta. Our results underline the safe use of Dinutuximab beta in the maintenance phase and also prove the feasibility of its use in r/r cases combined with Nivolumab.

However, continued research and further clinical trials with this combination therapy are essential and needed to improve survival rates and minimize the impact of treatment on long-term quality of life.

## Figures and Tables

**Table 1:** Characteristics of patients with neuroblastoma.

Characteristics	N (%)
Male	8 (53%)
Female	7 (46%)
Age (median, range)	1 year, 7 months–8 years
High-risk neuroblastoma	12 (80%)
*MYCN* amplification	2 (13.3%)

**Table 2:** A detailed description of our 5 patients who received immunotherapy.

	Patient 1	Patient 2	Patient 3	Patient 4	Patient 5
Age at diagnosis	8 months	5 years	7 years	3 years	7 months
Sex	Male	Female	Male	Male	Female
Tumor location	Right adrenal gland tu. paraaortic lymph node periorbital metastases *MYCN+*	Retroperitoneal tu. hepatic, retroperitoneal lymph node *MYCN–* inoperable	Right-sided paravertebral tu. mpx bone met. *MYCN–*	Retroperitoneal tu. paravertebral met. mpx bone met. *MYCN–* inoperable	Retroperitoneal tu. mesenterial mass mpx cranial bone met. *MYCN–* inoperable
Induction treatment	Chemotherapy (2 VP-Carbo, 4 CADO) Primary tu. resection Chemotherapy (Rapid COJEC) auto-HSCT radiotherapy	Chemotherapy (1 CO, Rapid COJEC, 4 TVD) auto-HSCT	Paravertebral tu. resection Chemotherapy (Rapid COJEC, 2 TVD) auto-HSCTradiotherapy	Chemotherapy (Rapid COJEC, 2 TVD) auto-HSCT radiotherapy	Chemotherapy (2 CO, 4 VP-Carbo, 4 CADO, Rapid COJEC, 2 TVD) auto-HSCT
Residual disease before immunoth.	Post-HSCT BM: 1–2% Post-RTx MR: CR	Post-HSCT BM: neg. Post-RTx MR: resid.tu Post-RTx mIBG: resid.tu	Post-HSCT BM: 5–10% Post-RTx MR: mpx bone met Post-RTx mIBG: neg.	Post-HSCT BM: neg. Post-RTx MR: resid.tu Post-RTx mIBG: resid.tu	Post-HSCT BM: neg. Post-HSCT MR: resid. tu. Post-HSCT mIBG: resid. tu.
Immunotherapy	Dinutuximab beta + Isotretinoin	Dinutuximabbeta + Isotretinoin	Dinutuximab beta + Isotretinoin	Dinutuximab beta + Isotretinoin	Dinutuximab beta + Cyclo-phosphamide + Irinotecan
Results after 5 cycles of Dinutuximab beta	CR	PR/SD	CR Relapse (4 months later)	PR/SD	PR
Further treatment and results	-	Nivolumab + Etoposide Progression	TEMIRI Alternative treatment Progression	Nivolumab + Etoposide Progression	Nivolumab + Etoposide Progression
Further treatment after progression—ongoing treatments	-	TEMIRI + Dinutuximab beta	-	TEMIRI + Dinutuximab beta	Nivolumab + TEMIRI + Dinutuximab beta
Latest follow-up results	CR	PR/SD	End-stage, progressive disease	PR/SD	PR/SD

## Data Availability

We confirm that neither the manuscript nor any parts of its content are currently under consideration for publication with or published in another journal. The data presented in this study are available on request from the corresponding author due to privacy, legal and ethical reasons.
